# Cobinamide, a Vitamin B_12_ Analog, Attenuates Benzo[a]pyrene and Pyrene Toxicity Through Selective Redox Modulation

**DOI:** 10.3390/toxics14050439

**Published:** 2026-05-15

**Authors:** Anirudh Kalyanaraman, Connor B. Stauffer, Weirui Gao, Tong Zhong, Alexandra Nguyen, Darren E. Casteel, Renate B. Pilz, Gerry R. Boss, Hema Kalyanaraman, John Tat

**Affiliations:** Department of Medicine, University of California, La Jolla, San Diego, CA 92093, USA; akalyanaraman@ucsd.edu (A.K.); cstauffer@ucsd.edu (C.B.S.); weg006@ucsd.edu (W.G.); tozhong@ucsd.edu (T.Z.); alexqnguyen1220@gmail.com (A.N.); dcasteel@health.ucsd.edu (D.E.C.); rpilz@health.ucsd.edu (R.B.P.); gboss@health.ucsd.edu (G.R.B.)

**Keywords:** benzo[a]pyrene, pyrene, reactive oxygen species (ROS), oxidative stress, cobinamide, antioxidant therapy, toxicity mitigation

## Abstract

Polycyclic aromatic hydrocarbons (PAHs) are common environmental contaminants formed during the incomplete combustion of organic material. Their persistence, bioaccumulation, and metabolic activation contribute to mutagenic and cytotoxic outcomes. Among these are benzo[a]pyrene (B[a]P), the most studied PAH and a benchmark compound for PAH carcinogenicity, and pyrene, a PAH whose urinary metabolite 1-hydroxypyrene is widely used as a biomarker of PAH exposure. B[a]P undergoes CYP1A1-mediated oxidation to generate reactive oxygen species (ROS) via epoxide and quinone redox cycling, whereas pyrene produces ROS primarily through pyrene-quinone redox cycling. We investigated cobinamide, a vitamin B_12_/cobalamin analog with potent antioxidant properties, for mitigating benzo[a]pyrene- and pyrene-induced injury. In H9C2 rat embryonic cardiomyoblasts and A549 human lung epithelial cells exposed to B[a]P (10 μM) or pyrene (10–100 μM), cobinamide (5–10 μM) attenuated PAH-induced reductions in cell number in both models, while in H9C2 cells, it also attenuated decreases in metabolic activity and reduced apoptosis. Cobinamide also returned JNK/p38 phosphorylation to near baseline levels, decreased DNA and protein oxidation and DNA strand breaks. Transcriptionally, cobinamide suppressed inflammatory (*TNF-α*, *IL-1β*, and *IL-6*) and oxidative stress genes (*HMOX1* and *NOX4*), while enhancing oxidative response (*SOD2*) and xenobiotic metabolism (*CYP1A1*). In *Drosophila melanogaster* exposed to 5 mM B[a]P/pyrene, 2 mM cobinamide improved survival and fully restored locomotion, outperforming cobalamin (minimal benefit) and *N*-acetylcysteine (partial rescue). Spectroscopic analyses showed no direct cobinamide-PAH binding. These findings demonstrate that cobinamide efficiently limits ROS-mediated PAH injury through redox modulation while preserving xenobiotic metabolism, suggesting its potential therapeutic use to mitigate PAH-induced toxicity.

## 1. Introduction

Polycyclic aromatic hydrocarbons (PAHs) are a class of organic compounds contain-ing multiple fused benzene, primarily generated during the incomplete combustion of organic matter [1,2,3]. The United States Environmental Protection Agency (EPA) recognizes 16 PAHs as priority pollutants because of their prevalence, capacity to bioaccumulate in aquatic organisms and enter the food chain, and their known or suspected carcinogenic properties [4].

One of the most extensively studied PAHs is benzo[a]pyrene (B[a]P), and its metabolism is well established [5,6,7]. Its metabolic activation begins during phase I metabolism, where cytochrome P450 enzymes (CYP1A1 and CYP1B1) oxidize B[a]P to B[a]P-epoxide [8,9]. This intermediate is hydrolyzed by epoxide hydrolase to form B[a]P-7,8-dihydrodiol, which can then undergo further CYP-mediated oxidation to generate B[a]P-diol epoxide (B[a]PDE). B[a]PDE is a highly mutagenic metabolite capable of forming covalent adducts with DNA, thereby initiating carcinogenic processes [10].

In addition to direct DNA damage, B[a]P metabolism can generate reactive oxygen species (ROS), which can oxidize and thereby damage DNA. During electron transfer in P450-mediated metabolism of B[a]P, incomplete reduction of oxygen can produce ROS such as superoxide anion (O_2_·^−^), hydrogen peroxide (H_2_O_2_), and hydroxyl radicals (·OH) [11]. These ROS can oxidatively damage DNA, proteins, and lipids. In parallel, a radical cation pathway of B[a]P metabolism involving P450 peroxidase activity forms DNA-reactive radical cations that are rapidly converted to quinones such as B[a]P-1,6-dione and B[a]P-3,6-dione. These quinones can redox cycle and generate additional ROS, contributing to oxidative injury [12].

Induction of P450 enzymes by PAHs occurs through activation of the aryl hydrocarbon receptor (AhR), a cytosolic ligand-activated transcription factor of the basic helix-loop-helix/Per-Arnt-Sim (bHLH/PAS) family that plays a central role in regulating xenobiotic metabolism [13,14,15]. In its inactive state, AhR is in the cytoplasm bound to chaperone proteins, including heat shock protein 90 (Hsp90), p23, and aryl hydrocarbon receptor-associated protein 9 (ARA9). Upon ligand binding, AhR dissociates from its chaperones, translocates to the nucleus, and dimerizes with the aryl hydrocarbon receptor nuclear translocator (ARNT) [16,17]. This heterodimer binds to xenobiotic response elements (XREs/AhREs) in DNA, activating transcription of genes such as *CYP1A1* and *CYP1B1* [18].

Pyrene, another EPA priority pollutant, is commonly used as a biomarker of PAH exposure via its urinary metabolite 1-hydroxypyrene [19,20,21]. Pyrene also activates the AhR/CYP1A1 pathway but is less toxic than B[a]P [22]. Its phase I metabolism yields 1,6- and 1,8-pyrenequinones that undergo NADPH:quinone oxidoreductase (NQO1)-dependent redox cycling, generating O_2_·^−^, H_2_O_2_, and ·OH [23]. Notably, unlike B[a]P, which can form stable DNA adducts via its bay-region epoxide, pyrene lacks this structure and induces genotoxicity primarily through ROS-mediated mechanisms, rather than direct DNA binding.

Because B[a]P and pyrene can stimulate ROS production, antioxidants (e.g., vitamin C, vitamin E, curcumin, fisetin, and lycopene) have been tested against B[a]P and pyrene toxicity and have shown some benefit in cultured cells and animal models [24,25,26,27,28]. Whether these drugs would be effective in humans is unclear, particularly since clinical trials with antioxidants have shown limited benefit in conditions where oxidative stress clearly contributes to the underlying pathophysiology. The reason for the lack of clinical efficacy of antioxidants may be that only agents with reaction rates above ~1 × 10^8^ M^−1^s^−1^ can meaningfully enhance the high endogenous antioxidant capacity [29].

Cobinanide (Cbi) is a vitamin B_12_/cobalamin (OHCbl) analog missing the 5,6-dimethylbenzimidazole ribonucleotide (DBZ) group (Supplemental Figure S1). The DBZ group is a crucial lower axial ligand to the central cobalt atom in the corrin ring. Lacking the DBZ group confers cobinamide with unique chemical properties compared to cobalamin. (1) Cobinamide binds two ligands instead of one. (2) The cobalt atom has a higher affinity for ligands in the trans position without the bulky, sterically hindering DBZ group [30]. (3) Cobinamide is more soluble in aqueous solutions than cobalamin, and by lacking the labile phosphodiester bond, is more stable than cobalamin [31,32]. And, (4) the cobalt atom is more readily reducible (+270 mV vs. −40 mV standard potential) [33,34]. This enhanced redox potential enables rapid superoxide scavenging, with cobinamide in the +3 and +2 oxidation states reacting at 1.1 × 10^8^ and 1.9 × 10^8^ M^−1^ s^−1^, respectively [35].

The high reactivity makes cobinamide a promising antioxidant candidate. In this study, we tested cobinamide’s ability to mitigate the cellular and organismal toxicity of B[a]P and pyrene, including oxidative stress, apoptosis, and functional impairment.

## 2. Materials and Methods

### 2.1. Chemicals

Cobinamide was prepared by base hydrolysis of cobalamin (Nutrakey Industries, Qingdao, China) and purified using reversed-phase resins [36]. The purity of each batch was confirmed by high-performance liquid chromatography and mass spectrometry, and only batches exceeding 98% purity were used [37]. In this work, the term “cobinamide” is used generically, without reference to axial ligands, and all experiments were conducted with cobinamide in the +3 oxidation state. Additional chemicals include benzo[a]pyrene (catalog #B1760; Millipore, Burlington, MA, USA), pyrene (catalog #185515; Sigma Aldrich, Burlington, MA, USA), and *N*-acetylcysteine (catalog #A9166; Sigma Aldrich).

### 2.2. Cell Culture

Rat H9C2 embryonal cardiomyoblasts (catalog #CRL-144; American Type Culture Collection, Manassas, VA, USA) and human A549 lung adenocarcinoma cells (catalog #CCL-185; American Type Culture Collection) were grown in Dulbecco’s modified Eagle’s medium supplemented with 25 mM glucose, 10% fetal bovine serum, and 1% penicillin-streptomycin. H9C2 cells model PAH-associated cardiovascular risk, as epidemiological data link airborne PAH exposure to myocardial injury [38,39,40], while A549 cells are a human-relevant lung epithelial cell model because PAH exposure has been linked to human pulmonary diseases [41].

### 2.3. Cell Counting

A total of 5 × 10^5^ H9C2 or A549 cells were seeded per well in 12-well tissue culture plates (catalog #07-200-82; Corning Costar, Corning, NY, USA) overnight. Selected wells were then treated with 10 µM B[a]P or 100 µM pyrene. All wells received a final concentration of 1% dimethyl sulfoxide (DMSO) because B[a]P and pyrene were made in DMSO. After 24 h, selected wells were co-treated with an antioxidant (i.e., cobinamide, cobalamin, or N-acetylcysteine) at the indicated concentrations (i.e., 5 µM or 10 µM). Following another 24 h of incubation, cells were detached from the plate using TrypLE Express (catalog #12604; Gibco, Waltham, MA, USA) and counted using a TC-20 Automated Cell Counter (BioRad, Hercules, CA, USA).

### 2.4. Cellular Metabolic Activity Assay

H9C2 cells were seeded in 96-well tissue culture plates (catalog #FB012931; FisherBrand, Pittsburgh, PA, USA) overnight. Selected wells were treated with 10 μM B[a]P or 100 μM pyrene for 24 h, followed by 10 μM cobinamide for 24 h. Cells were assessed for metabolic activity by following the reduction of 3-(4,5-dimethylthiazol-2-yl)-2,5-diphenyltetrazolium bromide (MTT) to formazan over 2 h. The formazan product was solubilized and quantified by absorbance at 540 nm in a Syngery 2 Microplate Reader (BioTek, Winooski, VT, USA).

### 2.5. Immunofluorescence Staining

H9C2 cells were cultured on glass coverslips in 24-well plates (catalog #GS83.1836N; BioPioneer, San Diego, CA, USA). Selected wells were then treated with 10 µM B[a]P or 10–100 µM pyrene. All wells received a final concentration of 1% DMSO. After 24 h, selected wells received 10 µM cobinamide for 24 h. Cells were fixed in situ in 3.7% paraformaldehyde and permeabilized with 0.2% Triton X-100 in phosphate-buffered saline (PBS). They were processed as described previously using antibodies against 8-OH-dGuo (catalog #SC393871, Santa Cruz Biotechnology, Dallas, TX, USA), nitro-tyrosine (catalog # cat. 9691S; Cell Signaling, Danvers, MA, USA), p-histone H2AX/γ-H2AX (catalog #2577; Cell Signaling), and cleaved caspase-3 (catalog #9661S, Cell Signaling) [35,42,43]. After incubation with the primary antibody, samples were incubated with either a Texas-Red- (for 8-OH-dGuo and γ-H2AX) (catalog #111095003; Jackson Immunoresearch, West Grove, PA, USA) or fluorescein isothiocyanate- (for nitro-tyrosine and cleaved caspase-3) (catalog #111095003 and catalog #115095003; Jackson Immunoresearch) conjugated secondary antibody, and nuclei were counterstained with Hoechst 33342 dye (catalog #H3570; Thermo Fisher Scientific). The fully stained samples were viewed using a Keyence BZ-X700 fluorescence microscope (catalog #972080; Keyence Corporation of America, Itasca, IL, USA) and analyzed using ImageJ 1.54k.

### 2.6. Immunoblotting

Cells were grown on 12-well plates overnight and then treated with 10 µM B[a]P. Following 24 h of exposure to B[a]P, cells were treated with 10 µM Cbi for 24 h. Cells were harvested, and samples were subjected to polyacrylamide gel electrophoresis followed by transfer to a nitrocellulose membrane. Phosphorylation of JNK and p38 MAPK were evaluated using anti-pJNK (pThr183/pTyr185) (catalog #9251S; Cell Signaling) and total JNK (catalog #9252S; Cell Signaling) antibodies and anti-pp38 MAPK (pThr180/pTyr182) (catalog #9215S; Cell Signaling) and total p38 MAPK (catalog #9212S; Cell Signaling) antibodies.

For protein carbonylation, the extracts were incubated with 2,4-dinitrophenylhydrazine (DNPH) prior to electrophoresis using a 1X DNPH solution provided in the OxyBlot™ Protein Oxidation Detection Kit, according to the manufacturer’s instructions, and carbonylated proteins were detected using an anti-dinitrophenylhydrazone antibody (catalog #S7150; MilliporeSigma, Burlington, MA, USA) [43]. The signal was normalized to β-actin (catalog #SC47778; Santa Cruz Biotechnology). The blots were incubated with a horseradish peroxidase (HRP)-conjugated goat anti-rabbit secondary antibody (catalog 111-035-144; Jackson ImmunoResearch). Band intensity was determined by densitometric scanning using a Li-Cor Odyssey instrument over a range where band intensity was linear.

### 2.7. Quantitative Reverse Transcription-PCR

Cells were grown on 6-well plates overnight, treated with 10 µM B[a]P for 24 h before 10 µM Cbi was added. Total RNA was isolated using Trizol reagent (catalog #TR 118; Molecular Research Center, Cincinnati, OH, USA), reverse-transcribed using iScript cDNA synthesis kit (catalog #1708891; Bio-Rad), and PCR was performed using a MX3005P real-time PCR detection system (Agilent Technologies, Inc., Santa Clara, CA, USA) with Brilliant II SYBR Green Mix (catalog #A25742; Thermo Fisher Scientific). All primers were intron-spanning (except for *GAPDH* RNA) and were tested with serial complementary DNA dilutions. Relative changes in messenger RNA (mRNA) expression were analyzed using the comparative 2^−ΔΔCt^ method, with *GAPDH* RNA serving as an internal control. Primers were obtained from Eton Biosciences, Inc. Primers were obtained from Integrated DNA Technology. Primers for *CYP1A1* were (forward) 5′ GTCCTAGAGAACACTCTTCAGTTCA 3′ and (reverse) 5′ TAACCACCCAGAATCCAAGGC 3′. Primers for *HMOX1* were (forward) 5′ TAAGACCGCCTTCCTGCTC 3′ and (reverse) 5′ TGCAGAGGTAGTATCTTGAACC 3′. Primers for *IL-1β* were (forward) 5′ CTCACAGCAGCATCTCGACAAGAG 3′ and (reverse) 5′ TCCACGGGCAAGACATAGGTAGC 3′. Primers for *IL-6* were (forward) 5′ ACTTCCAGCCAGTTGCCTTCTTG 3′ and (reverse) *IL-6* (reverse) 5′ TGGTCTGTTGTGGGTGGTATCCTC 3′. Primers for *NOX4* were (forward) 5′ ACATCCACCAGATGTTGGGC 3′ and (reverse) 5′ CTTCTGTGATCCGCGAAGGT 3′. Primers for *GAPDH* were (forward) 5′ TTGTGCAGTGCCAGCCTC 3′ and (reverse) 5′ CTTGCCGTGGGTAGAGTCA 3′. Primers for *SOD2* were (forward) 5′ GCTGGCCAAGGGAGATGTTA 3′ and (reverse) 5′ TGTGATTGATATGGCCCCCG 3′. And, primers for *TNF-α* were (forward) 5′ ATGGGCTCCCTCTCATCAGTTCC 3′ and (reverse) 5′ GCTCCTCCGCTTGGTGGTTTG 3′.

### 2.8. Assessment of Ligand Binding to Cobinamide

The binding of Cbi in the +3 oxidation state to benzo[a]pyrene, pyrene, or potassium cyanide was assessed on a Kontron 960 dual-beam ultraviolet-visible spectrophotometer (Nova Biotech, El Cajon, CA, USA) using quartz cuvettes. Samples were placed in the sample cuvette, and DMSO was placed in the reference cuvette to blank the spectrum. Cobinamide (12.5 µM) and the ligands (50 µM benzo[a]pyrene, 50 µM pyrene, or 50 µM potassium cyanide) were prepared in DMSO. For each experiment, the spectrum of cobinamide alone and of the ligand alone was recorded, followed by measurement of a mixture containing both cobinamide and the ligand after a 2 min incubation at room temperature. DMSO was used as the reference.

### 2.9. Drosophila melanogaster Survival Study

*Oregon-R* strain *Drosophila melanogaster* (fruit flies) were reared on standard fly food supplemented with yeast. For survival assays, a 2 × 2 cm gauze was impregnated with vehicle (final concentration: 5% *w*/*v* sucrose, 1% Tween-40, and 3% DMSO), 2 mM cobinamide in vehicle, 5 mM B[a]P in vehicle, 5 mM pyrene in vehicle, or 2 mM cobinamide combined with B[a]P or pyrene in vehicle. 15 flies of various ages and both sexes were placed in each glass vial containing a treated gauze. Survival was recorded daily for four days, and surviving flies were transferred each day to a fresh glass vial with a newly impregnated gauze. Six independent experiments were performed.

### 2.10. Drosophila melanogaster Negative Geotaxis Assay

Flies of various ages and both sexes were exposed to the indicated experimental condition for 24 h. Survivors were anesthetized with CO_2_ using an anesthetic flow bed (catalog #21-177; Genesee Scientific, El Cajon, CA, USA), and 10 flies per condition were randomly selected. Individual flies were placed in separate 9.5 cm × 2.2 cm polystyrene vials and allowed to recover at room temperature for 20 min prior to testing. Each vial was gently tapped to displace the fly to the bottom, and the time required for the fly to climb 9 cm vertically was recorded. A maximum of 180 s was assigned to flies that did not complete the climb. Three independent experiments were performed, with 10 flies per condition.

### 2.11. Statistical Analysis

Statistical significance of normalized cell count data was assessed using Brown–Forsythe and ANOVA tests to account for unequal variances. Quantitative data (normalized when appropriate) from MTT, immunofluorescence, and immunoblot dosimetry, and RT-qPCR assays were analyzed by one-way ANOVA with Šidák’s multiple comparisons correction. *Drosophila melanogaster* survival curves were evaluated using two-way ANOVA with Dunnett’s test, while negative geotaxis data were analyzed by one-way ANOVA with Šidák’s test. All data were plotted in GraphPad Prism 10 (GraphPad Software Inc., San Diego, CA, USA) and expressed as (normalized) mean ± standard deviation (SD). *p*-values: * *p* ≤ 0.05, ** *p* ≤ 0.01, *** *p* ≤ 0.001, and **** *p* ≤ 0.0001; ns stands for not significant.

### 2.12. Schematics

Artificial intelligence (AI), specifically Google Gemini 3.1 Flash Image (Nano Banana 2), was employed to generate visual representations of key elements. These included sources of PAHs (e.g., cigarette smoke and fossil fuel combustion), relevant cellular structures (e.g., proteins and genes), and depictions of the experimental models (cell-based assays and *Drosophila melanogaster*), with attention to avoid copyright concerns. Custom prompts were designed for each concept to guide image creation. The resulting AI-generated visuals were organized, annotated, and integrated into a cohesive figure using a digital design software (Canva, https://www.canva.com).

## 3. Results

### 3.1. Cobinamide Rescues Mammalian Cells from B[a]P- and Pyrene-Induced Toxicity

**Figure 1:**
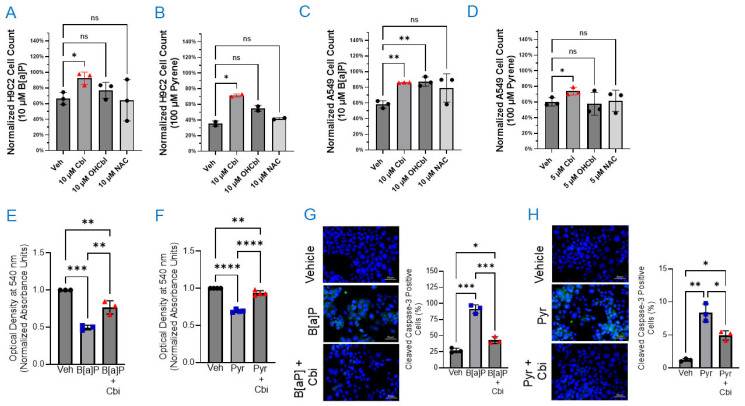
Rescue of B[a]P- and pyrene-induced toxicity by cobinamide in mammalian cells. H9C2 (**A**,**B**) or A549 cells (**C**,**D**) were treated with 10 μM B[a]P (**A**,**C**) or 100 μM pyrene (Pyr, **B**,**D**) for 24 h; cobinamide (Cbi), cobalamin (OHCbl), or *N*-acetylcysteine (NAC) were then added at the indicated concentrations for an additional 24 h, after which cells were harvested and counted. Data were normalized to vehicle (Veh)-, cobinamide-, cobalamin-, or *N*-acetylcysteine-treated controls, set at 100%, and represent the mean ± SD of *N* = 2–3 independent experiments, each performed in duplicate. Statistical comparison of PAH-treated cells versus PAH plus antioxidant-treated cells was performed using the Brown–Forsythe test followed by ANOVA with adjustment for unequal variances. (**E**,**F**) H9C2 cells were treated with 10 μM B[a]P (**E**) or 100 μM pyrene (**F**) for 24 h, followed by 10 μM cobinamide for 24 h, after which metabolic activity was assessed by MTT assay. Optical density was measured at 540 nm using a microplate reader. Data were normalized to vehicle control and represent the mean ± SD of *N* = 3–4 independent experiments. Statistical analysis was performed by two-way ANOVA with Šídák’s multiple comparisons test. (**G**,**H**) H9C2 cells were treated with 10 μM B[a]P (**G**) or 10 μM pyrene (**H**) for 24 h, followed by 10 μM cobinamide for 24 h, after which cleaved caspase-3 was assessed by immunofluorescence. Fluorescence microscopy images show cleaved caspase-3-positive cells (green) and DNA (Hoechst, blue) at 20× magnification. Quantification of cleaved caspase-3-positive cells is shown in the accompanying panels and represents the mean ± SD of *N* = 3 independent experiments. Scale bar represents 50 μm. Differences were evaluated by two-way ANOVA with Šídák’s multiple comparisons test. Statistical significance is indicated as follows: *p*-values: * *p* ≤ 0.05, ** *p* ≤ 0.01, *** *p* ≤ 0.001, and **** *p* ≤ 0.0001; ns stands for not significant.

H92C cells were treated with either 10 μM B[a]P or 100 μM pyrene for 48 h, with the indicated antioxidant added during the last 24 h. B[a]P significantly reduced the number of H9C2 cells to ~67% of baseline, which was partially restored to ~92% of baseline by cobinamide treatment. Neither cobalamin nor *N*-acetylcysteine yielded significant effects on cell rescue (Figure 1A). Pyrene significantly reduced H9C2 cell number to ~35% of baseline; this was increased to ~70% of baseline by cobinamide treatment. Again, neither cobalamin nor *N*-acetylcysteine showed significant cell rescue (Figure 1B). In A549 cells, 10 μM B[a]P reduced the cell number to ~58% of baseline; both cobinamide and cobalamin significantly increased cell number, ~86% and ~87%, respectively, while *N*-acetylcysteine was without effect (Figure 1C). Pyrene reduced the A549 cell count to ~60% of baseline; cobinamide increased cell number to ~74%, while neither cobalamin nor *N*-acetylcysteine had an effect (Figure 1D).

A549 cells were included to assess whether the effects of B[a]P, pyrene, and antioxidant treatment were consistent in a human epithelial cell line in addition to rat H9C2 cardiomyoblasts, providing an initial evaluation of generalizability (Figure 1C, D). Comparable directional responses were observed in both cell types. Subsequent experiments were conducted primarily in H9C2 cells to enable more detailed mechanistic studies in a non-tumorigenic, cardiac-relevant model, as cardiomyocytes are established targets of PAH-induced oxidative stress and cytotoxicity.

To determine whether the observed reduction in cell number was associated with changes in metabolic activity and/or apoptosis, we performed two separate experiments. First, we evaluated cellular metabolic activity by using an MTT reduction assay as an indicator of viable cell metabolic function. B[a]P treatment lowered MTT reduction by ~50%; cobinamide significantly increased metabolic activity to ~77% of baseline values (Figure 1E). Pyrene decreased MTT reduction by ~30%, with cobinamide significantly increasing metabolic activity to ~94% of baseline values (Figure 1F). Second, we assessed cellular apoptosis. B[a]P exposure induced a ~3.5-fold increase in cleaved caspase-3 levels relative to baseline, which was attenuated to ~1.8-fold above baseline with cobinamide co-treatment; apoptotic signals remained significantly elevated compared to vehicle control (Figure 1G). Similarly, pyrene treatment resulted in a ~6.9-fold increase in cleaved caspase-3, which was reduced to ~4.3-fold above baseline in the presence of cobinamide and remained significantly higher than vehicle-treated cells (Figure 1H). Cobinamide’s inability to fully reverse apoptosis will be addressed in the Discussion.

### 3.2. Cobinamide Protects H9C2 Cells from PAH-Induced Oxidative Stress and DNA Damage

**Figure 2:**
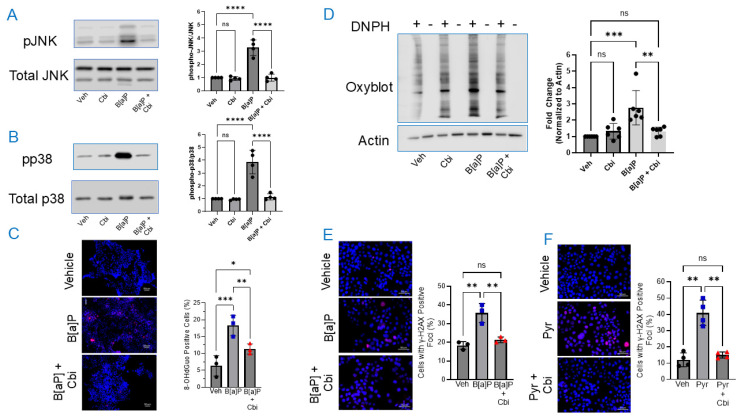
Cobinamide protects H9C2 cells from PAH-induced oxidative stress and DNA damage. (**A**,**B**) Stress-activated MAP kinases JNK (**A**) and p38 (**B**) were assessed by immunoblotting pJNK and pp38 relative to total JNK and p38. Representative immunoblots are shown for each condition, quantification of band intensities across *N* = 4 independent experiments is presented in the accompanying bar graph. (**C**) 8-hydroxy-deoxyguanosine (8-OH-dGuo), a marker of oxidative DNA damage, was assessed by immunofluorescence microscopy. Representative immunofluorescence images are shown for each condition. Fields were randomly chosen, and 100 cells were counted. The percentage of 8-OH-dGuo-positive cells in each field across *N* = 3 independent experiments is presented in the accompanying bar graph. Scale bar represents 50 μm. (**D**) Global protein oxidation was assessed using the Oxyblot^TM^ method. Protein bands were quantified by densitometry (ImageJ) and normalized to actin loading controls. A representative immunoblot is shown; quantification of band intensities across *N* = 6 independent experiments is presented in the accompanying bar graph. (**E**,**F**) DNA double-strand breaks were evaluated by γ-H2AX-positive foci using immunofluorescence microscopy. Representative immunofluorescence images are shown for each condition. A field was randomly chosen, and 100 cells were counted. Foci were quantified per cell; data represent the mean ± SD of *N* = 3 independent experiments for the B[a]P study (**E**) and *N* = 4 for the pyrene study (**F**). Scale bar represents 50 μm. All assays were performed in H9C2 cardiomyoblasts exposed to 10 μM B[a]P for 48 h, with 10 μM cobinamide (Cbi) added during the last 24 h. Data were normalized to vehicle-treated controls. Statistical analysis for all experiments was performed by two-way ANOVA with Šídák’s multiple comparisons test. Statistical significance is indicated as follows: *p*-values: * *p* ≤ 0.05, ** *p* ≤ 0.01, *** *p* ≤ 0.001, and **** *p* ≤ 0.0001; ns stands for not significant.

To explore the mechanisms underlying PAH-induced cytotoxicity and the protective effects of cobinamide, we assessed markers of oxidative stress, following the same protocol as for assessing cytotoxicity: cells were incubated for 48 h with B[a]P, and cobinamide was added during the last 24 h. Stress-activated mitogen-activated protein kinases (MAPKs), including JNK and p38, play central roles in cellular responses to environmental toxicants by mediating oxidative stress, inflammation, and apoptosis [44,45]. To determine whether these pathways are activated by PAH and modulated by cobinamide, we evaluated phosphorylated JNK (pJNK) and p38 (pp38) in H9C2 cells. B[a]P induced a robust activation of JNK-pJNK, which increased ~3.2-fold relative to vehicle control. Cobinamide effectively suppressed this induction, restoring pJNK to near baseline, while cobinamide alone had little effect on JNK phosphorylation (Figure 2A). Similarly, B[a]P markedly increased p38 phosphorylation, with pp38 levels rising ~3.7-fold relative to vehicle control; cobinamide eliminated the activation, reducing pp38 to ~1.18-fold of vehicle, while having no effect by itself (Figure 2B).

To evaluate oxidative injury in B[a]P-treated cells, we assessed DNA oxidation by immunofluorescence imaging of 8-oxodeoxyguanosine (8-oxo-dGuo)-positive cells and global protein oxidation using the Oxyblot system. B[a]P caused about a 2.5-fold increase in 8-oxo-dG-positive H9C2 cells relative to vehicle control, with cobinamide partially attenuating this effect, reducing 8-oxo-dGuo levels to ~1.5-fold above vehicle; however, DNA oxidation remained significantly elevated compared to vehicle control cells (Figure 2C). Cobinamide’s inability to fully reverse DNA oxidation will be addressed in the Discussion. B[a]P also markedly increased protein oxidation in H9C2 cells, resulting in a ~2.8-fold increase relative to vehicle-treated cells; cobinamide substantially suppressed the protein oxidation to ~1.3-fold of vehicle, while having no effect by itself (Figure 2D).

To further assess PAH-induced DNA damage, due both to oxidative stress and B[a]P’s propensity to bind to DNA and form adducts [46], and to pyrene’s indirect genotoxic effects via ROS induction, we measured γ-H2AX-positive foci as an indicator of DNA strand breaks. B[a]P almost doubled γ-H2AX foci relative to vehicle-treated H9C2 cells; cobinamide reduced γ-H2AX foci to near baseline values (Figure 2E). Pyrene produced about a 3.4-fold increase in γ-H2AX foci compared to vehicle, with cobinamide once again reducing foci to baseline values (Figure 2F).

Having established that cobinamide reduces B[a]P- and pyrene-induced oxidative stress, we sought to determine whether cobinamide’s effect was mediated solely through antioxidant activity or also through direct binding, and thereby neutralization, of PAHs. Spectrophotometric assays showed no evidence of B[a]P or pyrene binding to cobinamide; the spectral changes at wavelengths <425 nm were almost certainly due to the intrinsic absorbance of B[a]P and pyrene, and this will be addressed in the Discussion (Supplemental Figure S2). As a positive control, we show a clear spectral shift on adding potassium cyanide to cobinamide (Supplement Figure S2 Inset C). The data indicate that cobinamide’s protective effect is likely mediated via antioxidant activity rather than PAH sequestration.

### 3.3. Cobinamide Modulates PAH-Induced Transcriptional Responses Linked to Xenobiotic Metabolism, Inflammation, and Redox Homeostasis in H9C2 Cells

**Figure 3:**
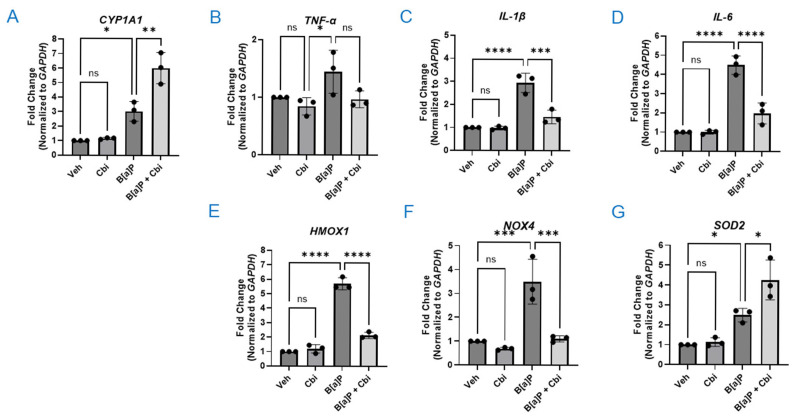
Cobinamide modulates PAH-induced transcriptional responses linked to xenobiotic metabolism, inflammation, and redox homeostasis in H9C2 cells. (**A**–**G**) Gene expression was assessed by RT-qPCR for: *Cyp1a1* (**A**), the pro-inflammatory cytokines *TNF-α*, *IL-1β*, and *IL-6* (**B**,**C**,**D**, respectively), and the antioxidant/redox-related genes *HMOX1*, *NOX4*, and *SOD2* (**E**,**F**,**G**, respectively). All assays were performed in H9C2 cardiomyoblasts exposed to 10 μM B[a]P for 48 h, followed by 10 μM cobinamide (Cbi) added during the last 24 h. Data were normalized to *GAPDH* RNA, and relative gene expression in vehicle-treated cells was set to 1. Data were normalized to vehicle-treated controls and represented the mean ± SD, *N* = 3 independent experiments. Statistical analysis for all experiments was performed by two-way ANOVA with Šídák’s multiple comparisons test. Statistical significance is indicated as follows: *p*-values: * *p* ≤ 0.05, ** *p* ≤ 0.01, *** *p* ≤ 0.001, and **** *p* ≤ 0.0001; ns stands for not significant.

Similar to previous work [47], we found that B[a]P induced *CYP1A1* ~3.0-fold relative to vehicle (Figure 3A). *CYP1A1* encodes the cytochrome P450 enzyme CYP1A1 that oxidizes B[a]P, and its expression is known to be suppressed by ROS [48,49]. Consistent with these latter results, cobinamide combined with B[a]P increased *CYP1A1* expression to ~6.0-fold above the vehicle control (Figure 3A). We will address this interesting finding in the Discussion. Among pro-inflammatory cytokines, B[a]P upregulated *TNF-α*, *IL-1β*, and *IL-6* by ~1.5-, ~3.0-, and ~4.5-fold, respectively; cobinamide largely mitigated the induction of all three cytokines, reaching significance for *IL-1β* (~1.5-fold) and *IL-6* (~2.0-fold) (Figure 3B–D). Within redox-related genes, B[a]P induced *HMOX1*, *NOX4*, and *SOD2* by ~5.7-, ~3.5-, and ~2.5-fold, respectively; cobinamide substantially reduced *HMOX1* (~2.2-fold) and *NOX4* (~1.1-fold) expression, while further increasing *SOD2* expression to ~4.3-fold of baseline (Figure 3E–G). The decrease in *NOX4* and increase in *SOD2* indicate that cobinamide simultaneously reduced ROS production while increasing antioxidant capacity. Cobinamide alone did not significantly affect the expression of any of the studied genes.

### 3.4. Cobinamide Ameliorates PAH-Induced Mortality and Neuromuscular Impairment in Drosophila melanogaster

To evaluate whether cobinamide’s protective effects in H9C2 cells extend to a whole animal, we used *Drosophila melanogaster* (fruit flies) as an in vivo model of PAH toxicity. *D. melanogaster* is increasingly employed in drug discovery and toxicology research due to its low maintenance cost, rapid reproduction, and ease of genetic/chemical manipulation, enabling assessment of mortality and functional deficits as a complement to cellular studies [50,51].

**Figure 4:**
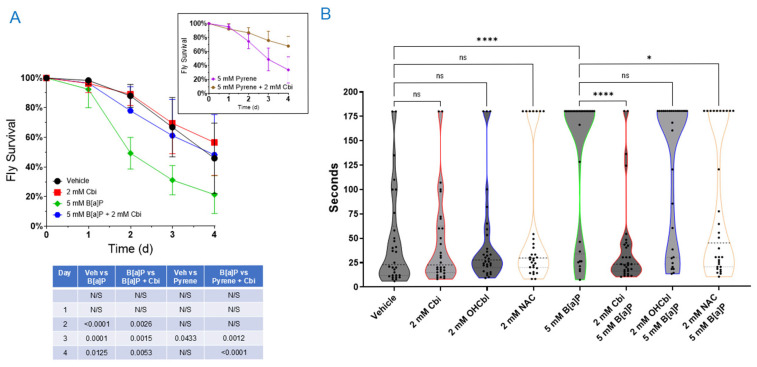
Cobinamide ameliorates PAH-induced mortality and neuromuscular impairment in *Drosophila melanogaster. Oregon-R* strain *Drosophila melanogaster* were reared on standard fly food supplemented with yeast. (**A**) Gauze squares were impregnated with vehicle (final concentration: 5% *w*/*v* sucrose, 1% Tween-40, and 3% DMSO), 2 mM cobinamide (Cbi) in vehicle, 5 mM B[a]P in vehicle, 5 mM pyrene in vehicle, or 2 mM cobinamide combined with each PAH in vehicle. 15 flies were placed in each vial containing a treated gauze. Survival was recorded daily for four days, with surviving flies transferred each day to fresh vials with newly impregnated gauze. Data are expressed as the mean ± SD. Each symbol represents data from an individual experiment. Statistical analysis was performed by two-way ANOVA with Dunnett’s multiple comparisons test. (**B**) Flies were exposed to the same conditions as in Panel A for 24 h, with cobalamin and *N*-acetylcysteine as additional antioxidative conditions. Survivors were anesthetized with CO_2_, and 10 flies from each condition were randomly selected for testing. Each fly was placed in a separate vial, allowed to recover at room temperature for 20 min, and then assessed for climbing performance. The time required to climb 9 cm vertically was recorded, with a maximum of 180 s assigned to those who could not complete the entire 9 cm climb. Data are expressed as the mean ± SD. Each symbol represents an individual fly. Statistical analysis was performed by one-way ANOVA with Šídák’s multiple comparisons test. Statistical significance is indicated as follows: *p*-values: * *p* ≤ 0.05, and **** *p* ≤ 0.0001; ns stands for not significant.

Flies of various ages and both sexes were exposed to 5 mM B[a]P or pyrene, with or without cobinamide, and survival was monitored over four days (Figure 4A). B[a]P caused a marked decrease in fly survival relative to vehicle-treated controls, with significant mortality evident from the second day and persisting through day four, indicating rapid and pronounced toxicity. In contrast, pyrene produced a more delayed effect, with a significant reduction in survival appearing only on day three, indicating lower overall toxicity compared with B[a]P. Co-treatment with 2 mM cobinamide significantly improved survival for both PAHs, with protection observed from day two to four for B[a]P and on days three and four for pyrene. Notably, a decline in survival was also observed in vehicle- and cobinamide-only flies. However, survival in these control groups remained comparable and did not account for the PAH-induced mortality patterns observed. This effect will be addressed in the Discussion.

In parallel, we assessed locomotor function in the flies using a negative geotaxis assay (Figure 4B). This method is a sensitive measure of neuromuscular health and overall physiological performance that detects oxidative stress-induced deficits, as shown in paraquat-treated *Drosophila* exhibiting ROS-mediated neurodegeneration and climbing impairment [52] and in DJ-1 *Drosophila* mutants displaying heightened locomotor dysfunction under oxidative stress [53]. We found that flies exposed to vehicle, cobinamide, cobalamin, and *N*-acetylcysteine all climbed normally, with mean times of ~45–60 s required to climb 9 cm. In contrast, B[a]P exposure markedly delayed climbing, with flies requiring an average of ~130 s to reach the target, reflecting significant neuromuscular dysfunction. Co-treatment with cobinamide restored climbing performance to baseline values (~42 s). Cobalamin co-treatment yielded minimal improvement (~123 s), whereas *N*-acetylcysteine showed partial yet significant rescue (~83 s). Thus, B[a]P impairs locomotor function in fruit flies that cobinamide restores, whereas other antioxidants provide limited or partial protection.

## 4. Discussion

Benzo[a]pyrene and pyrene are widely encountered PAHs that induce oxidative stress, DNA damage, and inflammation in exposed tissues, contributing to carcinogenic [4] and negative cardiovascular [38,39,40] and pulmonary [41] outcomes. Building on prior work showing that cobinamide is a potent, multifunctional ROS/RNS (reactive nitrogen species) scavenger with superior kinetics and solubility compared with conventional antioxidants [35], we asked whether cobinamide could attenuate B[a]P- and pyrene-induced toxicity in mammalian cells and in an in vivo *Drosophila* model, and whether cobinamide’s protective effects are primarily attributable to redox modulation rather than altered PAH metabolism or binding.

We found that both B[a]P and pyrene reduced cell number in H9C2 and A549 cells, with a parallel reduction in metabolic activity and increased apoptosis in H9C2 cells. Cobinamide provided consistently superior protection across models, while cobalamin showed only partial rescue in A549 cells with B[a]P (Figure 1C) and failed against pyrene in H9C2 cells. Cobalamin’s minimal protection likely reflects ROS scavenging during the second 24 h treatment window—after the initial AhR-driven PAH metabolism has generated the oxidative burst—but is constrained by its established AhR antagonism, likely via the 5,6-dimethylbenzimidazole ribonucleotide, which suppresses AhR activation essential for efficient detoxification [54]. Unlike cobalamin, cobinamide lacks the 5,6-dimethylbenzimidazole ribonucleotide moiety and thus preserves AhR/CYP1A1 signaling (Figure 3A). The incomplete protection by cobinamide may be due to the experimental protocol of adding cobinamide during the last 24 h of a 48 h PAH exposure period. This was done to simulate a clinical scenario where treatment could be delayed for some time after PAH exposure, for example, in an isolated military burn pit.

We examined whether cobinamide acts mainly by modulating canonical stress response pathways and oxidative damage, or whether additional protection could arise from direct PAH binding and sequestration. We found that B[a]P strongly activated JNK and p38 and increased oxidative DNA and protein damage in H9C2 cells, whereas delayed addition of cobinamide largely normalized JNK/p38 phosphorylation, substantially reduced DNA oxidation and protein carbonylation, and lowered γ-H2AX foci to near baseline values. We found no evidence that cobinamide directly binds and sequesters B[a]P or pyrene (Supplemental Figure S2). The spectral changes that occurred at low wavelengths (<425 nm) on adding B[a]P or pyrene to cobinamide are explained by the intrinsic absorbance of the PAHs, and thus only the changes that were observed at higher wavelengths can reliably indicate cobinamide binding. Together, these results argue that cobinamide’s effects are mediated at least in part by antioxidant modulation of canonical stress response pathways, rather than by direct sequestration of B[a]P or pyrene.

PAHs are known to induce changes in gene expression at the transcriptional level. We examined (1) xenobiotic metabolism (*CYP1A1*) as a readout of PAH sensing and bioactivation, (2) pro-inflammatory cytokines (*TNF-α*, *IL-1β*, and *IL-6*) as markers of downstream inflammatory signaling, and (3) redox-regulatory genes (*HMOX1*, *NOX4*, and *SOD2*) as indicators of oxidative stress and endogenous antioxidant responses. We found that B[a]P exposure robustly induced *CYP1A1*, upregulated *IL-1β* and *IL-6* (with a more modest increase in *TNF-α*), and elevated *HMOX1*, *NOX4*, and *SOD2* expression, consistent with activation of aryl hydrocarbon receptor-dependent xenobiotic metabolism, inflammatory pathways, and redox stress responses.

The transcriptional responses observed with cobinamide co-treatment can be understood in the context of its antioxidant and modulatory effects: genes primarily induced by oxidative stress, including *HMOX1* and *NOX4*, and the pro-inflammatory cytokines *TNF-α*, *IL-1β*, and *IL-6* exhibited reduced expression when cobinamide was combined with B[a]P, likely reflecting its potent ROS-scavenging activity that diminishes the cellular stress signals driving these genes. In contrast, *CYP1A1*, a canonical xenobiotic-metabolizing enzyme, and *SOD2*, a mitochondrial antioxidant, were further elevated with cobinamide co-treatment.

B[a]P induced AhR-driven *CYP1A1* expression as expected [47]. The combination of B[a]P with cobinamide produced even higher *CYP1A1* expression than B[a]P alone, which initially appeared counterintuitive. However, this superinduction aligns with established negative feedback wherein CYP1A1-derived ROS (e.g., H_2_O_2_) inactivate nuclear factor I (NF-I) at the proximal promoter, hindering further *CYP1A1* transcription, thereby limiting detoxification [48,49]. By scavenging ROS, cobinamide relieves this redox repression, permitting maximal AhR activation while simultaneously suppressing the B[a]P-induced increases in *HMOX1*, *NOX4*, *TNF-α*, *IL-1β*, and *IL-6* expression and JNK/p38 activation.

Enhanced *SOD2* expression with cobinamide co-treatment suggests additional priming of mitochondrial antioxidant defenses beyond direct ROS scavenging. SOD2, the principal scavenger of mitochondrial superoxide, is regulated by transcription factors including Nrf2 (nuclear factor erythroid 2-related factor 2) and FOXO family members (e.g., FOXO3a) [55]. By mitigating ROS, cobinamide may indirectly activate Nrf2 via KEAP1 dissociation, enabling Nrf2 binding to the *SOD2* promoter and driving its transcription—a common adaptive response in oxidative stress models. Similarly, ROS reduction could preserve FOXO3a nuclear localization by limiting Akt-mediated phosphorylation, further upregulating *SOD2* transcription. Although we did not examine Nrf2, this mechanism aligns with cobinamide’s redox modulation and warrants future studies in PAH-induced lung/cardiac toxicity.

Altogether, cobinamide could be useful for PAH toxicity mitigation. Maximal CYP1A1 expression ensures rapid xenobiotic detoxification without PAH bioaccumulation, while ROS scavenging normalizes downstream stress responses.

In *Drosophila melanogaster*, we demonstrated that B[a]P (and, to a lesser extent, pyrene) markedly reduced survival and climbing performance, confirming PAH toxicity at the organismal level. Cobinamide co-treatment significantly improved survival and fully restored locomotor function. Cobalamin, which has intrinsic antioxidant properties, had no significant effect on locomotion, while *N*-acetylcysteine provided only partial locomotor improvement. In the survival studies, the toxicity observed in vehicle- and cobinamide-only flies may be related to the solvents required to deliver B[a]P. Due to its extremely low aqueous solubility arising from multiple benzene rings, B[a]P had to be initially dissolved in 3% Tween-40, diluted in 100% DMSO, and then further diluted in 5% *w*/*v* sucrose to the final 5 mM exposure concentration, the minimum concentration required to induce lethality. Both Tween-40 and DMSO are inherently toxic to flies and likely account for the baseline mortality in vehicle- and cobinamide-only groups. However, this background effect did not account for the distinct PAH-induced survival patterns observed. Importantly, this solvent-induced toxicity did not negatively impact neuromuscular health, at least during the 24 h negative geotaxis assay, as flies exposed for this duration climbed normally. However, longer exposures of 48 or 72 h revealed that surviving vehicle- and cobinamide-only flies lost climbing ability, suggesting that solvent effects accumulate over time and that shorter exposures are critical for accurately assessing locomotor performance.

**Figure 5:**
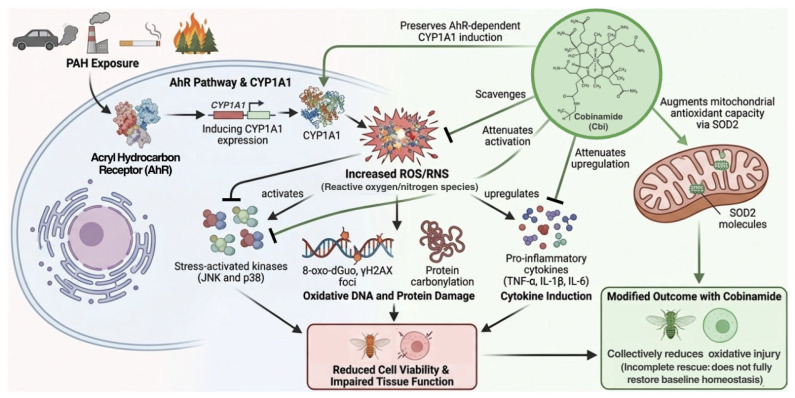
Proposed mechanism of cobinamide-mediated protection against PAH-induced oxidative injury. Polyaromatic hydrocarbon (PAH) exposure activates the aryl hydrocarbon receptor (AhR), inducing CYP1A1 expression and promoting metabolic activation that increases reactive oxygen and nitrogen species (ROS/RNS). Elevated ROS/RNS and stress-activated kinases (JNK and p38) lead to oxidative DNA and protein damage (8-oxo-dGuo, γH2AX, and protein carbonylation) and upregulation of pro-inflammatory cytokines (TNF-α, IL-1β, and IL-6), culminating in reduced cell and animal viability, and impaired locomotor function in the animal model. Cobinamide, a highly reactive, multifunctional ROS/RNS scavenger, attenuates toxicity by scavenging ROS/RNS and reducing stress-activated kinase activation and inflammatory signaling while preserving AhR-dependent CYP1A1 induction and augmenting mitochondrial antioxidant capacity via SOD2. These effects collectively reduce oxidative injury but do not fully restore baseline homeostasis. Arrows indicate activation, whereas T-shaped bars indicate inhibition. Note: the ROS-dependent feedback mechanism underlying CYP1A1 superinduction described in the Discussion is not explicitly depicted in this simplified schematic.

Based on our findings, we propose a model (Figure 5) in which the polyaromatic hydrocarbon benzo[a]pyrene and pyrene activate the AhR pathway and CYP1A1 expression to drive oxidative/inflammatory stress via elevated ROS/RNS, JNK/p38 phosphorylation, DNA/protein damage, and cytokine induction. Cobinamide, a highly reactive, multifunctional ROS/RNS scavenger, substantially attenuated toxicity across cellular and organismal models by scavenging reactive species, dampening stress-activated kinase/pro-inflammatory signaling, and enhancing mitochondrial antioxidant defenses via SOD2 upregulation while preserving PAH sensing.

## 5. Conclusions

This study demonstrates that cobinamide, a highly reactive, multifunctional ROS/RNS scavenger, substantially attenuated B[a]P- and pyrene-induced toxicity across cellular and organismal models by targeting oxidative stress-associated injury pathways while leaving PAH sensing intact. The observed protection was partial, consistent with a mechanism centered on redox modulation rather than direct effects on PAH metabolism or PAH-induced genotoxic injury. Cobinamide’s consistent superiority over cobalamin and *N*-acetylcysteine positions it as both a chemical tool for dissecting PAH-associated oxidative injury and a promising countermeasure for environmental toxicant exposure. Future studies in patient-derived organoids, mammalian models, and realistic PAH mixtures will be essential for clinical translation.

## 6. Limitations

A major limitation of this study was the reliance on H9C2 cells as the primary in vitro model. As a two-dimensional culture system, H9C2 cells cannot fully recapitulate the complex architecture, cell–cell interactions, and metabolic gradients present in tissues and organs. Similarly, while *Drosophila melanogaster* provided an excellent in vivo platform to assess survival and neuromuscular function, flies differ from mammals in metabolism, physiology, and xenobiotic processing. Thus, neither model fully captures mammalian systemic physiology or human exposure complexity. Nonetheless, the combined use of H9C2 cells and flies allowed us to rigorously evaluate cobinamide’s protective effects across both cellular and organismal contexts, providing complementary evidence that PAH-induced oxidative stress, apoptosis, and functional impairments can be mitigated. Future studies using three-dimensional organoids or mammalian models will be important to confirm and extend the beneficial effects of cobinamide against PAH-induced toxicity.

## Data Availability

The original contributions presented in this study are included in the article/Supplemental Material. Further inquiries can be directed to the co-corresponding authors (H.K. and J.T.).

## Supplementary Materials

The following supporting information can be downloaded at: https://www.mdpi.com/article/10.3390/toxics14050439/s1. Figure S1: Structures of cobalamin and cobinamide; Figure S2: Cobinamide does not bind B[a]P or pyrene but binds cyanide.
