# Supplementary material for: Cobinamide, a Vitamin B12 Analog, Attenuates Benzo[a]pyrene and Pyrene Toxicity Through Selective Redox Modulation
Source: Toxics. 2026 May 15;14(5):439. doi: 10.3390/toxics14050439 (PMC13211721; doi:10.3390/toxics14050439)
Supplement: Supplementary file 1 [file toxics-14-00439-s001.zip › toxics-4255442-supplementary.pdf]

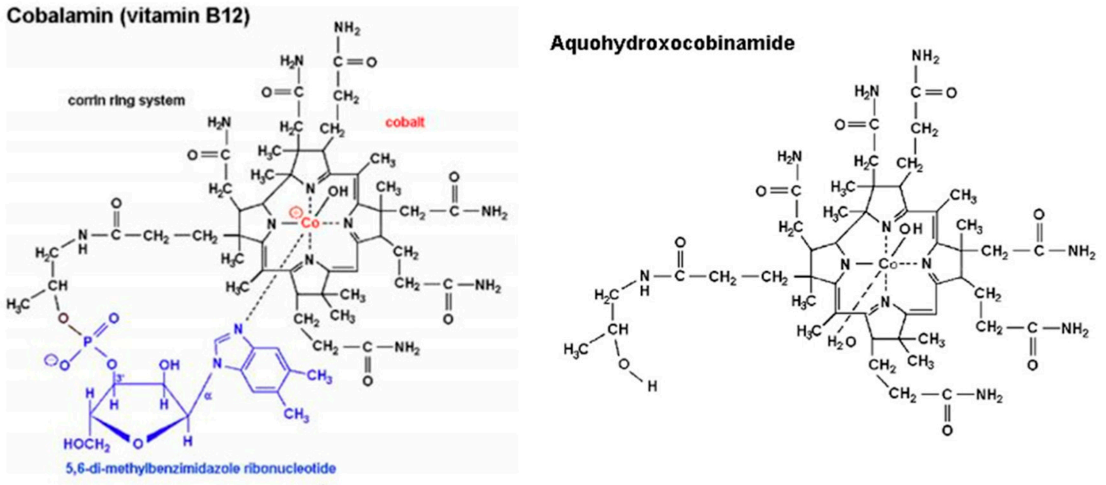

Supplementary Figure S1. Structures of cobalamin and cobinamide.

(A) The structure of cobalamin features a hydroxyl group (-OH) at the fifth coordination site and a 5,6-dimethylbenzimidazole ribonucleotide (DBZ) moiety (blue) at the sixth coordination site.

(B) The structure of cobinamide shows absence of the DBZ group present in cobalamin. Aquohydroxocobinamide forms when the sixth coordination site on cobinamide is occupied by a water (H_2O) molecule.

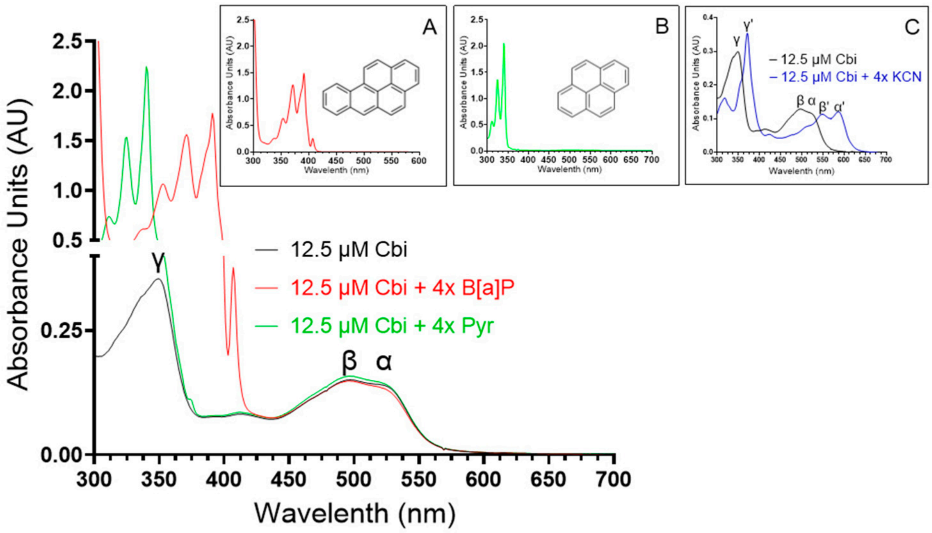

Supplementary Figure S2. Cobinamide does not bind B[a]P or pyrene but binds cyanide.

B[a]P, pyrene (Pyr), or potassium cyanide was added to an aqueous solution of 12.5 µM cobinamide (Cbi) at final concentrations up to fourfold the Cbi concentration. Ultraviolet-visible spectra were recorded from 300 to 700 nm. Cobinamide did not show appreciable spectral changes upon addition of B[a]P or pyrene, indicating a lack of interaction with the PAHs. In contrast, a fourfold excess of

cyanide produced a clear spectral shift under the same conditions (**Inset C**). (**Inset A**) Ultraviolet-visible spectrum of 12.5 μM B[a]P alone. (**Inset B**) Ultraviolet-visible spectrum of 12.5 μM pyrene alone.
